# Skin Commensal Fungus *Malassezia* and Its Lipases

**DOI:** 10.4014/jmb.2012.12048

**Published:** 2021-01-27

**Authors:** Minji Park, Sungmin Park, Won Hee Jung

**Affiliations:** Department of Systems Biotechnology, Chung-Ang University, Anseong 17546, Republic of Korea

**Keywords:** Lipase, *Malassezia*, *M. globose*, *M. restricta*, skin microflora

## Abstract

*Malassezia* is the most abundant genus in the fungal microflora found on human skin, and it is associated with various skin diseases. Among the 18 different species of *Malassezia* that have been identified to date, *M. restricta* and *M. globosa* are the most predominant fungal species found on human skin. Several studies have suggested a possible link between *Malassezia* and skin disorders. However, our knowledge on the physiology and pathogenesis of *Malassezia* in human body is still limited. *Malassezia* is unable to synthesize fatty acids; hence, it uptakes external fatty acids as a nutrient source for survival, a characteristic compensated by the secretion of lipases and degradation of sebum to produce and uptake external fatty acids. Although it has been reported that the activity of secreted lipases may contribute to pathogenesis of *Malassezia*, majority of the data were indirect evidences; therefore, enzymes’ role in the pathogenesis of *Malassezia* infections is still largely unknown. This review focuses on the recent advances on *Malassezia* in the context of an emerging interest for lipases and summarizes the existing knowledge on *Malassezia*, diseases associated with the fungus, and the role of the reported lipases in its physiology and pathogenesis.

## Introduction

*Malassezia*, an abundant genus of the fungal microflora found on human skin, is recognized as an opportunistic fungus owing to its association with various skin diseases [1-3]. The phylum, subphylum, class, order, and family of *Malassezia* are Basidiomycota, Ustilaginomycotina, Malasseziomycetes, Malasseziales, and Malasseziaceae, respectively [4]. Fourteen well-established species of *Malassezia* and four additional species have been identified to date. Out of the 18 species, 10 species (*M. restricta*, *M. globosa*, *M. arunalokei*, *M. sympodialis*, *M. dermatis*, *M. slooffiae*, *M. furfur*, *M. obtusa*, *M. japonica*, and *M. yamatoensis*) were isolated mainly from human skin, whereas the others are normally isolated from animal skin [5]. In the case of zoophilic species, *M. pachydermatis* is one of the most dominant fungal species isolated from companion animals, such as dogs [6]. *M. restricta* and *M. globosa* have been identified as the most predominant fungal species on human skin by several studies involving early culture-based, targeted polymerase chain reaction (PCR)-based, or recent skin mycobiome analysis-based studies [1, 7-9].

*Malassezia* seems to have evolved in niche specific manner and is well-adapted to the skin environment, wherein carbohydrates are restricted but lipids are abundant, a condition that favors the lipophilic characteristics of the fungus. *Malassezia* possesses multiple genes encoding lipases; hence, a number of studies have suggested that the secreted lipases might play an important role in not only the survival but also in the pathogenesis of the fungus in the hosts [10-12]. In this review article, we focused on the recent advances on *Malassezia* in the context of an emerging interest for lipases. We present the background information for the skin diseases associated with *Malassezia* and summarize the current knowledge on the fungal lipases.

## *Malassezia*-Associated Skin Diseases in Humans

Numerous studies that analyzed the fungal communities on human skin suggested *Malassezia* as the predominant fugal genus. Mycobiome analyses of samples collected from 14 different body sites of 10 healthy adults revealed that *M. restricta* and *M. globosa* were commonly isolated from the glabella, external auditory canal, and retroauricular crease; and the occiput, back, and inguinal crease; respectively [1]. Comparison of the fungal communities inhabiting skin between healthy adults and children revealed that *M. restricta* dominates at the sebaceous sites in adults rather than in children [9]. Furthermore, it was shown that the occiput, back, and manubrium exhibited *M. globosa* predominance, whereas *M. restricta* was predominant in the external auditory canal, retroauricular crease, and forehead [9]. Since *Malassezia* lives on the skin surface, it is usually associated with numerous skin diseases, such as seborrheic dermatitis/dandruff, atopic dermatitis, and pityriasis versicolor. Although various studies suggested that skin disorders and *Malassezia* are linked, our knowledge regarding the physiology and pathogenesis of *Malassezia* in skin diseases is still largely limited.

Seborrheic dermatitis and dandruff are the most common skin diseases worldwide. Seborrheic dermatitis, a chronic and inflammatory skin disease that occurs on the areas with a rich supply of sebaceous glands, including face, scalp, chest, and upper trunk, is characterized by scaling and redness. Dandruff is a mild form of seborrheic dermatitis and its pathological skin condition can be defined as abnormal scalp flakes accompanied by itching without inflammation [13]. The causal relationship of seborrheic dermatitis and dandruff with *Malassezia* has been shown: (1) effective treatment of the disorder following the use of antifungal drugs, (2) improvement of symptoms accompanied by quantitative reduction of *Malassezia*, and (3) recurrence of dandruff induced by *Malassezia* metabolite [14-17]. Besides, a number of large-scale sequencing analyses showed positive correlations between seborrheic dermatitis, dandruff, and *Malassezia* suggesting the fungus to be a potential disease causing agent.

Among the different species of *Malassezia*, *M. restricta* may play a critical role in the pathogenesis of seborrheic dermatitis and dandruff. Analysis of the fungal community in the scalp of the French population showed *M. restricta* as the most abundant fungal species on scalps with dandruff and scalps that were healthy. However, *M. restricta* was more abundant in the scalps with dandruff than that in healthy scalps and in the dandruff rich areas than in the non-dandruff areas [18]. Similar results were observed for the mycobiome analysis of the scalp of Chinese and Koreans subjects with and without dandruff, thereby verifying the disequilibrium of a balanced microbiome and the correlation between increased *M. restricta* population and dandruff [2, 19, 20]. A recent mycobiome and metagenome analyses involving the Indian female population revealed a significantly increased predominance of *M. restricta* over *M. globosa* in the swab samples from the scalps with dandruff compared to that in the healthy scalp, thus reiterating the critical role of *M. restricta* in dandruff [3]. Metagenome analysis showed enrichment of the N-glycan biosynthesis pathway of *M. restricta* in the scalp with dandruff compared to that in the healthy scalp. Since glycoprotein of the fungal cells is involved with adhesion to the host tissue [21], this pathway may be related to colonization of *M. restricta* on the scalp surface, thus further demonstrating the critical role of the fungus in dandruff [3]. Moreover, a mycobiome study on healthy scalps and scalps with dandruff revealed two different operational taxonomic unit levels of *M. restricta*, suggesting that distinct subspecies within the same species might play a different role in the disease [22].

Atopic dermatitis (AD) is a complex inflammatory disease caused by abnormal skin barrier function and immune response [23]. Owing to the defective epidermal barrier, skin with AD gets easily infected by the residential microflora, triggering the host immune responses. Among the resident microflora of the skin, *Malassezia* is thought to possess an antigenic property that induces type I hypersensitivity reaction in the skin. One of the hallmarks of AD is the elevated level of immunoglobulin E (IgE) in total serum of most patients, leading to IgE-mediated allergic reaction [24]. Several studies have reported hypersensitization to *Malassezia*-specific IgE in patients with AD. For example, a high proportion of AD patients were sensitized to *M. sympodialis* extract and recombinant *M. sympodialis* allergens, whereas patients with seborrheic dermatitis and healthy individuals exhibited no such sensitization [25]. Serum IgE from AD patients was more sensitized to *Malassezia* antigens [26]. A study reported positive correlation between *Malassezia*-specific IgE levels and disease severity in adult AD patients [27]. A study showed the increased levels of specific IgE against *M. restricta* compared to the other species of *Malassezia* in the sera of AD patients and suggested that *M. restricta* might also play an important role in AD [28]. In addition, a study that focused on the link between the allergens from *Malassezia* and AD showed that *M. globosa* allergen (MGL_1304), identified in sweat, induces the release of histamine from the basophils in AD patients but not in healthy individuals [29]. Moreover, the levels of MGL_1304-specific IgE in the sera of AD patients were significantly increased compared to those of the healthy individuals [30], and the activity of histamine release by MGL_1304 and its homolog Mala r 8 from *M. restricta* were higher than that of Mala s 8 produced by *M. sympodialis* [31]. These results imply that IgE-mediated sensitization against *Malassezia* and its allergens can act as a triggering factor for AD.

Pityriasis versicolor, characterized by hypo- or hyper-pigmentation in the seborrheic lesions of skin without inflammation, is one of the most common skin diseases caused by *Malassezia* infection of the stratum corneum. In general, pityriasis versicolor occurs frequently in adolescents and young adults with highly active sebaceous glands. Abundance of *Malassezia* in the lesions of patients with pityriasis versicolor is significantly increased compared to those in the unaffected skin sites of the patients or in healthy individuals, which suggests the etiological correlations between the disorder and the fungus [32, 33]. Although the exact role of *Malassezia* in pityriasis versicolor is not yet clear, the role of the metabolites produced by *Malassezia* in the pathogenesis has been demonstrated. Examples of these metabolites include indoles and indole derivatives derived from tryptophan by *Malassezia*, *M. furfur* in particular. Indirubin, malassezin, pityriacitrin, 6-formylindolo[3,2-b]carbazole, and indolo[3,2-b]carbazole generated by *M. furfur* serves as ligands of the aryl hydrocarbon receptor, which is a nuclear receptor belonging to the basic helix-loop-helix family of the transcription factor with implications in the association of the metabolites with pityriasis versicolor [34-36].

*Malassezia* is not only a part of the residential microflora of human skin, but it is also found in various other organs. Mycobiome analysis using stool samples from healthy individuals showed a high prevalence of *Malassezia*, especially *M. restricta* [37-40]. Furthermore, high prevalence of *M. restricta* in the water-lavage samples obtained from non-inflamed intestinal regions of patients with Crohn’s disease undergoing screening colonic endoscopy suggested the association of the fungal species with the disease [41]. A recent study also showed increased abundance of *Malassezia* in fecal samples from patients with pancreatic or colorectal cancer compared to those in the healthy control groups implying that the fungus might be linked to cancer in organs, as well as the diseases related to skin [42, 43]. Furthermore, a respiratory mycobiome study revealed the association between *Malassezia* and cystic fibrosis lung disease [44]. These findings imply that *Malassezia* plays a critical role in the human body than what is believed.

## The Potential Role of Lipases in the Pathogenesis of *Malassezia*

Skin is the largest organ of the human body, which form the first barrier against external environment. Lipids, the main constituents of the stratum corneum produced by the sebaceous glands, contribute significantly to the barrier function of skin. The lipid mixture within the stratum corneum primarily comprises ceramides, cholesterol, and fatty acids. Human sebum contains triglycerides, wax monoesters, squalene, and a small amount of cholesterol and cholesterol esters [45]. Given that *Malassezia* requires lipids for growth, the lipid-rich skin environment serves as a suitable habitat for the fungus. A cell culture-based study showed increased *Malassezia* population in adult than in children [46]; sebum level of the skin of the former was significantly high than that on the latter population [47]. Although culture-independent real-time PCR-based analysis and a mycobiome study revealed *Malassezia* as the predominant fungal genus on the adult skin, fungal communities were more diverse on the skin of children [8, 9].

Since species of *Malassezia* are unable to synthesize fatty acids, they must uptake external fatty acids as a nutrient source for survival. Genome analyses of multiple *Malassezia* species revealed the absence of the gene encoding fatty acid synthase [10, 12]. This lack of fatty acid synthesis ability is responsible for the lipid dependency of *Malassezia*, a characteristic which is compensated by the secretion of various lipases and degradation of sebum to produce and uptake external fatty acids [10]. Indeed, genome analyses of several *Malassezia* species showed significantly higher number of lipase encoding genes than that in other fungi [10-12].

Pathogenic microorganisms secrete enzymes with lipolytic activities to survive within the host. Among the different lipolytic enzymes, extracellular lipase, which primarily catalyzes the hydrolysis of triglycerides, is considered an important virulence factor [48, 49]. In this regard, several studies have suggested the important roles of lipases in microbial pathogenesis. For example, almost all clinically isolated bacterial pathogens, including *Staphylococcus aureus*, exhibited lipolytic activities [50, 51], and inhibition of chemotaxis and reduction of granulocytes mediated phagocytic killing of *S. aureus* cells by purified lipase from the bacteria [52, 53]. Moreover, a recent study showed that Geh lipase inhibits activation of innate immune cells, thereby verifying the critical roles of lipases in the pathogenesis of *S. aureus* [54].

Lipases also play an important role in fungal pathogenesis. For example, *Candida albicans* possesses at least 10 genes (*CaLIP1*–*CaLIP10*) encoding lipases. All the *CaLIP* genes, except *CaLIP7*, contain a signal peptide, implying that they produce extracellular lipases. Expression of *CaLIP5*, *CaLIP6*, *CaLIP8*, and *CaLIP9* during systemic candidiasis suggested their association with the pathogenesis of *C. albicans* [55]. Despite the temporal and stage-specific expression of *CaLIP* genes in general, *CaLIP5* and *CaLIP8* were predominantly expressed in the liver and kidney of infected mice, and *CaLIP4*, *CaLIP5*, and *CaLIP8* were expressed in at least 50% of the saliva specimens from patients with oral candidiasis [56]. Moreover, a *CaLIP8* deficient mutant with significantly reduced virulence in the murine intravenous infection model confirmed the importance of lipases in the pathogenesis of *C. albicans* [57]. Contribution of lipases in the fungal virulence was also suggested in other species of *Candida* such as *C. parapsilosis*, which possesses *CpLIP1* and *CpLIP2* genes encoding lipases. These lipases were required for intracellular survival of *C. parapsilosis* within the macrophages and reduced production of inflammatory cytokines in the host [58, 59].

Given the lipid-dependent characteristics of *Malassezia* and the crucial role of lipases in fungal survival on the skin, a large number of studies have been carried out to characterize the lipases in *Malassezia* (Table 1). *Malassezia* left the unsaturated fatty acids, oleic acid in particular, on the skin after consuming the saturated fatty acids in sebum, and it triggered dandruff formation in the dandruff susceptible individuals, the finding of which supports the role of lipases in the pathogenesis of *Malassezia* [60].

The lipase activity was first described in *M. furfur* [61, 62]. *MfLIP1*, encoding extracellular lipase, was also first identified and characterized in *M. furfur*. Recombinant MfLip1 enzyme expressed in *Pichia pastoris* exhibited hydrolytic activity against monoglycerides and showed highest activity at 40°C and pH 5.8 [63]. Elevated activity of an extracellular lipase in *M. furfur* with increased pH suggested that the environmental conditions critically influence the activity of the enzyme [64]. During the yeast to hyphal dimorphic transition of *M. furfur*, extracellular lipase activity was reported to be higher in hypha form than in the yeast from [65]. The same lipase activity was also observed in *M. globosa*. MgLip1 hydrolyzed mono- and diglycerides, but not triglycerides. Expression of *MgLIP1* on the human scalp demonstrated using reverse transcription-PCR suggested that lipase may play an important role in the survival of *M. globosa* on the host skin surface [66]. Another lipase, MgLip2 exhibited its optimal activity at 30°C and pH 5.0 [67]. A putative diacylglycerol-like lipase, MgMdl2, was also identified and characterized. Its recombinant form showed optimal activity at 15°C and pH 6.0. Similar to MgLip1, recombinant MgMdl2 also utilized mono- and diglycerides, but not triglycerides [68]. Interestingly, a structural study showed that amino acid substitutions, such as Phe278Ala and Glu282Ala converted the substrate specificity of MgMdl2 from mono- and diglycerides to triglycerides, suggesting the critical role of these two amino acid residues in substrate recognition [69]. Subsequently, genome analysis of *M. globosa* revealed that the fungus possesses at least 14 lipase encoding genes [11]. Thirteen of them were expressed in *P. pastoris* and the hydrolysis activity of each enzyme was analyzed against various substrates. Results revealed that MgLip1, MgMdl2, MgMdl3, MgMdl4, MgMdl5, and MgMdl6 are the Class 3 family lipases with specificity for mono- and diglycerides, whereas MgLip2, MgLip3, MgLip4, MgLip5, and MgLip7 are the LIP family lipases that hydrolyze all substrates including triglycerides, one of the key components in human sebum [70].

In *M. restricta*, initially 12 lipase genes were identified in the genome of the strain KCTC 27527; however, a recently resequenced genome analysis of the same strain revealed two additional lipase genes [12, 71]. Among the 14 lipases identified in *M. restricta*, three secretory lipases (MrLip1, MrLip2, and MrLip3) were purified and characterized. MrLip1 and MrLip2 were members of Class 3 family, and MrLip3 belonged to the LIP family of lipases. Similar to MgLip1 in *M. globosa*, MrLip1 and MrLip2 specifically degrade mono- and diglycerides. In contrast, MrLip3 hydrolyzes triglycerides as well as mono- and diglycerides [72]. MrLip1 exhibited optimal enzyme activity at 34°C and pH 5.0; conditions similar to those found on normal healthy skin surface. Furthermore, expression patterns of MrLip1 were well correlated with the optimal pH and temperature of the enzyme activity [73]. Our previous study showed that *MrLIP5*, which encodes the LIP family lipase, was expressed on the scalp of 53 out of 56 patients with severe dandruff, suggesting a possible association of MrLip5 with dandruff and pathogenesis of *Malassezia* [12]. Subsequent purification of MrLip5, and analysis of the gene expression levels and enzyme activity under different pH conditions revealed highest expression level and optimal enzyme activity at pH 7.0–8.0 [74]. Given that the pH increases in the diseased skin surface [75-77], our observation suggested that MrLip5 might play a major role in the pathogenesis of *Malassezia* on the diseased skin surface.

In addition to the possible pathological roles of secreted lipases in *Malassezia*, association between secreted lipases and mechanism of the action of an antifungal agent was suggested. Example includes treatment of *Malassezia* with the antifungal drug zinc pyrithione (ZPT). Clinical isolates of *M. restricta* treated with ZPT showed significantly reduced expression levels of several secreted lipases, including MrLip1, MrLip3, and MrLip5. This finding indicated that inhibition of lipase functions is one of the key mechanisms of action of ZPT; hence, the lipases secreted from *Malassezia* could be a potential target for antifungal drug [78].

## Concluding Remarks

**Fig. 1:**
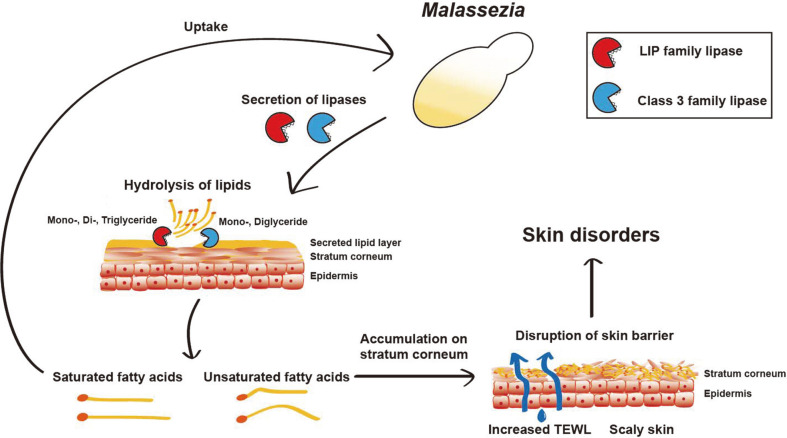
The potential role of lipases secreted by *Malassezia* in skin diseases. Lipases secreted by *Malassezia* decompose the human skin sebum-derived lipids, such as mono-, di-, and triglycerides, into saturated and unsaturated fatty acids. The saturated fatty acids are consumed by *Malassezia* for survival, whereas the unsaturated fatty acids accumulate on the stratum corneum. This accumulation might interfere with the permeability of the skin barrier, thereby leading to various skin disorders. TEWL: Transepidermal water loss.

Research on *Malassezia* has gained more attention owing to its association with not only diseases on the skin but also its presence in other organs of the human body. However, the roles of *Malassezia* in human body are still largely unknown. Existing studies suggest that lipases may play critical role in the survival and pathogenesis of *Malassezia* on the human skin surface. The lipases decompose sebum to free fatty acids. Saturated fatty acids are primarily consumed by *Malassezia*, whereas the unsaturated fatty acid, oleic acid in particular, remains on the stratum corneum and likely triggers disruption of the skin barrier function (Fig. 1) [17].

Similar to other pathogenic microorganisms, secreted lipases in *Malassezia* may also modulate or interfere with the host immune cell responses. However, to date, information on the roles of lipase in *Malassezia* is restricted to in vivo observation of lipase gene transcripts and *in vitro* analysis of purified enzyme activity. Genetic experiments using a mutant lacking the gene encoding lipase are certainly required to understand the role of lipases in physiology and pathogenesis of *Malassezia*. The gene knockout method utilizing *Agrobacterium tumefaciens*-mediated transformation is available for few *Malassezia* species, including *M. furfur*, *M. sympodialis*, and *M. pachydermatis* [79, 80]. However, a similar gene manipulation technique is yet to be developed for *M. restricta* and *M. globosa*, the two most dominant species of *Malassezia* in human body. Therefore, future studies are warranted to develop molecular genetic tools for increasing our understanding of the physiology and pathogenesis of *M. restricta* and *M. globosa* and to elucidate the role of lipases in *Malassezia* infections.

## Figures and Tables

**Table 1:** Lipases in different species of *Malassezia* that are commonly found on human skin.

Species	Predicted function from Pfam analysis (ID/accession)	Gene ID	Signal peptide	Optimal condition	Substrate	Reference
*M. restricta* KCTC 27527	Class 3 (PF01764)	MRET_0019 (*MrLIP1*)	O	pH 5, 34°C	Mono- and diglyceride	[12, 71-73]
		MRET_1032	×			[12, 71]
		MRET_2826 (*MrLIP4*)	O			[12, 71]
		MRET_3772	O			[12, 71]
		MRET_4032 (*MrLIP2*)	O		Mono- and diglyceride	[12, 71, 72]
		MRET_4356	×			[12, 71]
	LIP family (PF03583)	MRET_0930 (*MrLIP5*)	O	pH 7		[12, 71, 74]
		MRET_1179 (*MrLIP3*)	O		Mono-, di-, and triglyceride	[12, 71, 72]
		MRET_4098	O			[12, 71]
		MRET_4099	O			[12, 71]
	Partial alpha/beta-hydrolase lipase region (PF04083)	MRET_1507	×			[12, 71]
		MRET_4144	×			[12, 71]
	Putative serine esterase, Lipase-like (PF05057)	MRET_3765	×			[12, 71]
	GDSL-like Lipase/Acylhydrolase (PF00657)	MRET_3994	O			[12, 71]
*M. globosa*CBS 7966	Class 3 (PF01764)	MGL_0279	×			[11]
		MGL_0797 (*MgLIP1*)	O		Mono- and diglyceride	[11, 70]
		MGL_0798 (*MgMDL3*)	O		Mono- and diglyceride	[11, 70]
		MGL_0799 (*MgMDL2*)	O	pH 6, 15°C	Mono- and diglyceride	[11, 68, 70]
		MGL_0800 (*MgMDL4*)	O		Mono- and diglyceride	[11, 70]
		MGL_1311 (*MgMDL5*)	O		Mono- and diglyceride	[11, 70]
		MGL_1769	×			[11]
		MGL_3878 (*MgMDL6*)	O		Mono- and diglyceride	[11, 70]
	LIP family (PF03583)	MGL_1311 (*MgLIP4*)	O		Mono-, di-, and tri-glyceride	[11, 70]
		MGL_3507 (*MgLIP6*)	O		Diglyceride	[11, 70]
		MGL_4051 (*MgLIP7*)	O		Mono-, di-, and triglyceride	[11, 70]
		MGL_4052 (*MgLIP5*)	O		Mono-, di-, and triglyceride	[11, 70]
		MGL_4054 (*MgLIP2*)	O	pH 5, 30°C	Mono-, di-, and triglyceride	[11, 67, 70]
		MGL_4197 (*MgLIP3*)	O		Mono-, di-, and triglyceride	[11, 70]
	Partial alpha/beta-hydrolase lipase region (PF04083)	MGL_1975	×			[11]
		MGL_2531	×			[11]
	Putative serine esterase, Lipase-like (PF05057)	MGL_4063	×			[11]
	GDSL-like Lipase/Acylhydrolase (PF00657)	MGL_1366	O			[11]
*M. sympodialis* ATCC 42132	Class 3 (PF01764)	MSYG_1326	O			[81]
		MSYG_2002	×			[81]
		MSYG_2462	O			[81]
		MSYG_2628	O			[81]
		MSYG_3125	O			[81]
		MSYG_3126	O			[81]
		MSYG_4275	×			[81]
	LIP family (PF03583)	MSYG_2467	O			[81]
		MSYG_2468	O			[81]
		MSYG_2630	O			[81]
		MSYG_3257	O			[81]
	Partial alpha/beta-hydrolase lipase region (PF04083)	MSYG_0158	×			[81]
		MSYG_2613	×			[81]
	Putative serine esterase, Lipase-like (PF05057)	MSYG_3580	×			[81]
	GDSL-like Lipase/Acylhydrolase (PF00657)	MSYG_1283	×			[81]
